# Neutrophils phagocytosing fungal hyphae in urinary sediment

**DOI:** 10.1590/2175-8239-JBN-2019-0245

**Published:** 2020-12-21

**Authors:** José Antonio Tesser Poloni, Clotilde Druck Garcia, Liane Nanci Rotta, Constantin F. Urban

**Affiliations:** 1 Universidade do Vale do Rio dos Sinos, Escola de Saúde, São Leopoldo, RS, Brasil.; 2 Controllab, Rio de Janeiro, RJ, Brasil.; 3 Santa Casa de Misericórdia de Porto Alegre, Serviço de Nefrologia Pediátrica, Porto Alegre, RS, Brasil.; 4 Universidade Federal de Ciências da Saúde de Porto Alegre, Faculdade de Medicina, Porto Alegre, RS, Brasil.; 5 Universidade Federal de Ciências da Saúde de Porto Alegre, Departamento de Métodos Diagnósticos, Porto Alegre, RS, Brasil.; 6 Umeå University, Umeå Centre for Microbial Research, Department of Clinical Microbiology, Umeå, Sweden.

**Keywords:** Phagocytosis, Hyphae, Urinalysis, Neutrophils., Fagocitose, Hifas, Urinálise, Neutrófilos.

## Abstract

The Phagocytosis of fungal structures by neutrophils is a well-documented function of these immune cells. However, neutrophil phagocytosis of hyphal structures in the urine sediment is not usually observed during routine sample evaluation. This is a case of hyphal phagocytosis by neutrophils in the urine of a kidney allograft recipient patient.

## Resumo

A fagocitose de estruturas fúngicas por neutrófilos é uma função bem documentada destas células imunes. No entanto, a fagocitose de hifas por neutrófilos no sedimento urinário não é normalmente observada durante avaliação de rotina de amostras. Este é um caso de fagocitose de hifas por neutrófilos na urina de um paciente receptor de aloenxerto renal.

## Case

**Figure 1:**
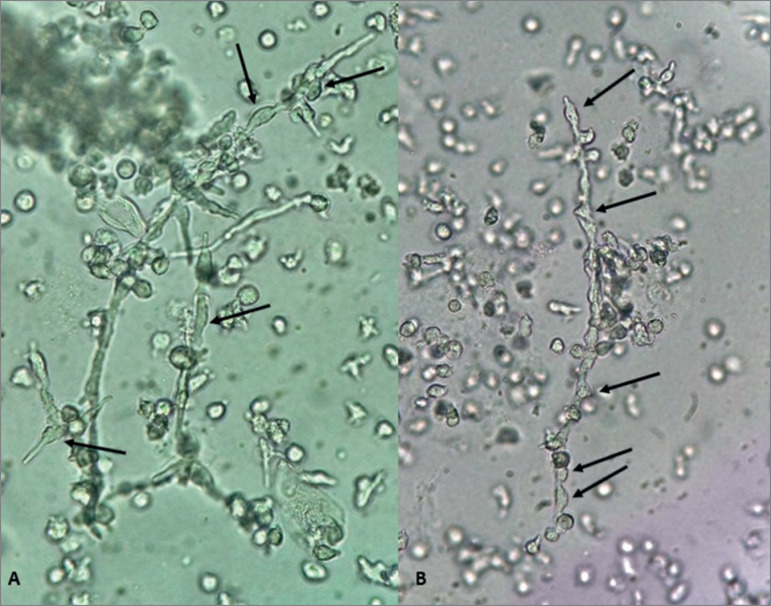
(A and B): Fresh and unstained urine sediment viewed in bright field microscopy. Original magnification 400x. At first glance, leukocytes are seen as enlargements of the fungal structure. However, moving carefully the micrometer of the microscope it is possible to note that there are cells (leukocytes) attached to hyphae.

**Figure 2:**
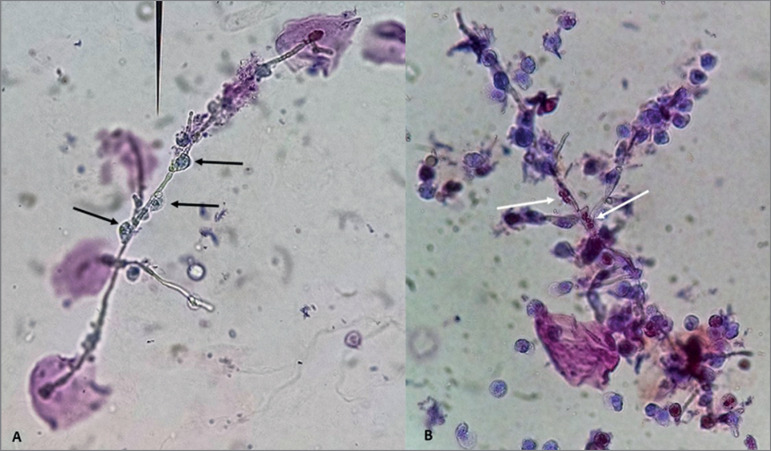
(A and B): Fresh urine sediment stained with Sternheimer-Malbin stain viewed in bright field microscopy. Original magnification 400x. The use of stain reveals the nuclear structure of the leukocytes and in some of them a trilobular nucleus is clearly seen (white arrows). Neutrophils are the most common leukocyte observed in urine samples and in this case they were firmly attach to hyphal structures.

An 8-year-old girl with malformation of the urogenital sinus and kidney dysplasia developed chronic kidney disease. Due to the loss of kidney function, a kidney transplantation became necessary and was subsequently performed. The patient was diagnosed with Biedl-Bardet syndrome, a condition that is characterized by polydactyly, obesity, retarded growth development, as well as genital and renal abnormalities. During consultation, vulvovaginitis was observed and the patient presented with mictional disorder resulting in abnormally frequent urination. Routine urinalysis by dipstick revealed the following: specific gravity 1,009, pH 6.0; hemoglobin traces, leukocyte esterase 2+, and nitrite positive; all remaining tests were negative. Urine sediment analysis presented the following results: epithelial cells 7-8/high power field (HPF), leukocytes 19-29/HPF, and erythrocytes 1-2/HPF. In addition, renal tubular epithelial cells and decoy cells (BK polyomavirus infected cells) were detected. Interestingly, fungal hyphae were spotted with firmly attached polymorphonuclear leukocytes, apparently attempting to perform phagocytosis, which, to the best of our knowledge, was never reported during routine urinalysis previously (Figures 1 and 2). Despite the positive nitrite test, uroculture was negative for bacteria, whereas a culture test in specific medium for Candida species turned out positive for *Candida albicans*. Subsequently, the patient was topically treated with Nystatin, which alleviated the symptoms.

*Candida albicans* stimulates recruitment of neutrophils and can be phagocytosed by these cells. *C. albicans* is the major agent of fungal infections in humans, with immunocompromised hosts particularly sensitive to this microorganism. *C. albicans* can modify their structures from budding cells to filamentous hyphae, a characteristic that can contribute to the effectiveness of the infection control. The immune system acts against Candida infection performing phagocytosis of fungal structures with the cell membrane recognizing the shape of the pathogenic agent.

Fungi are able to modify their structures depending on the necessity and conditions of the site of infection. Hyphae more commonly invade and penetrate tissues, while yeasts act on the spread of the microorganism. Yeasts use the blood vessels for dissemination^1^^-^ 6.

Neutrophils are one of the main immune cells that act in the control of pathogenic agents in an infection site. The mechanism of destruction of the engulfed particles is done by antimicrobial proteins and reactive oxygen species. The phagocytic process starts when phagocytosis receptors recognize specific molecular patterns. Ligand-receptor binding stimulates the cells to engulf the recognized microbe.

To eliminate yeasts, neutrophils sequestrate these fungal structures in phagosomes. Reactive oxygen species (ROS) and elastase are released within the phagosomes improving the process of yeast destruction. Hyphae on the other hand, are more challenging for neutrophils because they are large and cannot be internalized. In this case, azurophilic granules deliver their content into the nucleus, triggering chromatin decondensation and release of neutrophil extracellular traps (NETs). NETs have a contribution in the process of immobilization and destruction of extracellular pathogens but with the disadvantage of causing tissue injury^7^^-^ 10. Investigation of NET formation in clinical specimens is the next step in the comprehension of neutrophil response against fungal agents within the urinary system.

In this urine sample, leukocytes (neutrophils, as trilobular nuclei were clearly seen in the stained sample) were observed covering virtually the whole fungal structure. The phagocytic cells stretched and deformed considerable involving the fungal structure (hyphae) in the attempt to engulf it. It is reasonable to assume that the neutrophils within the specimen were not merely attached to the fungal cells, but were in a phagocytic process.

We conclude that this finding in a urinary sediment sample is an *in vivo* indication of phagocytosis of *C. albicans* by neutrophils within the urinary tract, a previously unreported finding. It indicated the action of the immune system against this kind of pathogenic agent. This observation highlights the wide spectrum of structures in urine sediment analysis and helps microscopy technicians to properly recognize this kind of urinary finding.
